# A new approach for calculating microalgae culture growth based on an inhibitory effect of the surrounding biomass

**DOI:** 10.1007/s00449-021-02550-6

**Published:** 2021-04-16

**Authors:** Sun-Hwa Jung, Christopher McHardy, Cornelia Rauh, Alexander Jahn, Giovanni Luzi, Antonio Delgado, Rainer Buchholz, Christoph Lindenberger

**Affiliations:** 1 Department of Mechanical Engineering and Environmental Engineering, OTH Amberg-Weiden, Kaiser-Wilhelm-Ring 23, 92241 Amberg, Germany; 2 Department of Food Biotechnology and Food Processing Engineering, Technische Universität Berlin, Königin-Luise-Str. 22, 14195 Berlin, Germany; 3 German Engineering Research and Development Centre LSTME Busan, Busan, South Korea; 4 Department of Bioprocess Engineering, FAU Busan Campus, Friedrich-Alexander University, Busan, South Korea

**Keywords:** Algae cultivation, Numerical methods, Growth kinetics, Inhibitory effects

## Abstract

Ever since the potential of algae in biotechnology was recognized, models describing the growth of algae inside photobioreactors have been proposed. These models are the basis for the optimization of process conditions and reactor designs. Over the last few decades, models became more and more elaborate with the increase of computational capacity. Thus far, these models have been based on light attenuation due to the absorption and scattering effects of the biomass. This manuscript presents a new way of predicting the apparent growth inside photobioreactors using simple models for enzymatic kinetics to describe the reaction between photons and the photosynthetic unit. The proposed model utilizes an inhibition kinetic formula based on the surrounding biomass to describe the average growth rate of a culture, which is determined by the local light intensities inside the reactor. The result is a mixed-inhibition scheme with multiple inhibition sites. The parameters of the new kinetic equation are replaced by empirical regression functions to correlate their dependency on incident light intensity and reactor size. The calibrations of the parameters and the regression functions are based on the numerical solutions of the growth rate computed with a classical Type II model. As a final verification, we apply the new equation in predicting the growth behavior of three phototrophic organisms in reactors of three different sizes.

## Introduction

The boom of algal biotechnology in the last decades arises — to a certain extent — from the political pressure driving the search for a green alternative to fossil fuels. In the context of the energy revolution, bioenergy increased the public awareness of the possibilities to utilize microalgae.

Besides bioenergy, microalgae biomass contains various products ranging from little-processed animal feed to dietary supplements and chemical building blocks to active pharmaceutical substances [1]. The general notion of processing algae is a simple idea of combining CO_2_, light, and some nutrients. Nutrient supply and mass transfer from the gaseous phase are well-known from traditional bioprocess engineering. However, light supply still poses challenges to the scientific community; it is the missing link to scale-up and efficient process optimization. To overcome this problem, on the one hand, new inventions for efficient light supply have been proposed [2]. On the other hand, mathematical models are being developed to achieve a better understanding of the relation between culture growth and light attenuation inside the reactor.

Early models tried to connect light attenuation to the growth behavior within a reactor system. They were based on calculating the mean light intensity in photobioreactors, correlating it to Monod kinetics [3, 4]. Due to the nonlinear relationship between growth and light and the exponential-like decay of the light intensity with increasing penetration depth, these simple models are only reliable when all process parameters remain in the same range. Several mistakes come along with scale-up and alternating process parameters when the calculation of the average growth is based on the average light intensity, as the average light intensity does not correlate to one specific light distribution inside the rector. Those mistakes can be overcome by first calculating the light intensity profile in the reactor. In a second step, the growth rates at a single location are correlated with the light intensities. The volumetric average of all local growth rates represents the apparent growth of the culture. A slightly different approach is based on dividing the reactor into different light zones, therefore, reducing the calculation time by avoiding computations of dark zones [5]. A more complete and sophisticated approach involves calculating the light supply of single cells moving inside the reactor by solving the full three-dimensional radiative transfer equation and the complete three-dimensional set of Navier–Stokes equations [6, 7]. For the correlation between light and growth kinetics, it is ubiquitous to use simple kinetics according to Monod or extended ones by light inhibition [4, 8, 9]. With increasing computational power it became possible to perform numerical simulations on molecular levels, computing the interaction between photons and the photosynthetic units [10]. Those approaches require a crosslink to molecular changes inside the cell — primarily increase and decrease of pigments — as they are influencing the efficiency of the adsorption process and light attenuation [11]. In 2013, Béchet [12] classified the single approaches of predicting growth in photobioreactors into Type I, Type II, and Type III models. Type I models predict the growth of the whole culture using average light intensity. Type II models average local growth rates inside the reactor and Type III models extend Type II ones by including the photosynthesis rate [12]. Another — yet contrary — trend in predicting the performance of photobioreactors corresponds to the Hartmanis principle, which states a correlation between the simplicity of a formula and its usefulness for application, by introducing simple empirical methods that allow a precise assessment of productivity and growth [13].

Different from the light attenuation dependent kinetic models approaches based on conservation of mass and energy assume a homogeneous thermodynamical system, e.g., a photobioreactor. Therefore, the biomass yield of a system can be correlated to the enthalpy and the Gibbs energy using the stoichiometry of biomass growth and the energy of the absorbed photons [14]. In this article, we are introducing a new approach for calculating the apparent growth inside photobioreactors. Instead of calculating the light attenuation by the surrounding biomass, the apparent growth rate is expressed by a kinetic function that considers the inhibition of the surrounding biomass. The presence of biomass inhibits the growth rate by mutual shading, that is, by absorbing and scattering the light that enters the reactor. The underlying strategy is to reproduce the asymmetric sigmoid relation between biomass concentration and the apparent growth rate. The characteristic shape and the degree of asymmetry of the function are mainly dependent on the incident light intensity and the reactor size. The calibration of the new derived logistic formula to numerical solutions of a classical Type II model facilitates the calculation of growth rates in cylindrical reactors of different sizes and variations of the incident light, by only changing the input of those values. The new approach is a good alternative to Type II models — even when reactor diameters change.

## Theory

In perfectly mixed cultures, the growth can be determined by solving the mass balance equation:

1 $$\frac{\mathrm{d}XV}{\mathrm{d}t}={\dot{V}}_{\mathrm{in}}{X}_{\mathrm{in}}{-\dot{V}}_{\mathrm{out}}{X}_{\mathrm{out}}+(\overline{\mu }-\dot{D})XV.$$

${\dot{V}}_{in}$ and ${\dot{V}}_{out}$ indicate the volume flow rate that enters and leaves the reactor, respectively. $V$ is the reaction volume, $X$ is the biomass concentration, $\stackrel{-}{\mu }$ is the average growth rate of the culture, which depends on the light supply and $\dot{D}$ is the uniform death rate or respiration decay rate. Besides, ${X}_{in}$ and ${X}_{out}$ denote the biomass concentrations that enter and leave the vessel, respectively. In the case of a simple batch process with constant volume where no biomass enters or leaves the reactor during the process, Eq. 1 reduces to:

2 $$\frac{\mathrm{d}X}{\mathrm{d}t}=X (\overline{\mu }-\dot{D}).$$

Assuming that the photosynthetic response of algae is much faster than the mixing in the reactor, the apparent growth rate is equal to the growth rate averaged over the cross-sectional area of the vessel,

$$\stackrel{-}{\mu }\;=\;\frac{{\mu }_{max}}{\pi {r}^{2}}\underset{0}{\overset{2\pi }{\int }}\underset{0}{\overset{r}{\int }}\frac{I(b,X)}{I(b,X)+{K}_{M}}b\mathrm{d}b\mathrm{d}\varphi,$$

where $r$ is the radius of the reactor, ${K}_{M}$ is the half-saturation constant,$b$ is the radial direction and $\varphi$ is the polar angle. Integrating the previous equation with respect to $\varphi$ gives:

3 $$\stackrel{-}{\mu }\;=\;2\frac{{\mu }_{max}}{{r}^{2}}\underset{0}{\overset{r}{\int }}\frac{I(b,X)}{I(b,X)+{K}_{M}}b\mathrm{d}b.$$

Influences of the top and the bottom walls of the cylindrical vessel on the biomass growth rate are neglected. By assuming that light attenuation inside the vessel is mainly caused by cell absorption and neglecting scattering effects, one can compute the light distribution $I$ at each point inside the reactor employing the Lambert–Beer law:

4 $$I(p,X)={I}_{0}{e}^{-p\alpha X}.$$

**Fig. 1:**
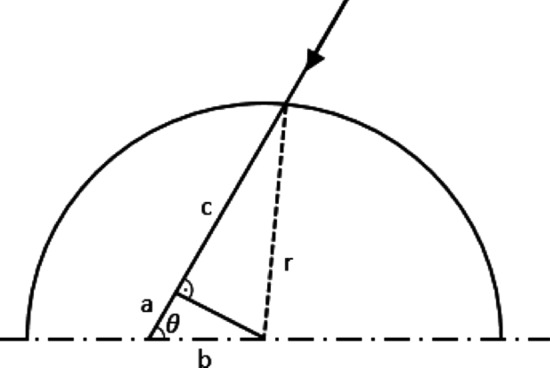
Schematic representation of the half cross-section of a cylindrical reactor

Here, $\alpha$ is the absorption cross-section, ${I}_{0}$ is a constant light intensity at the reactor surface (incident light intensity) and $p$ is the length of the light path, which reads according to Fig. 1 [15]:

5 $$p\left(\theta ,b\right)=a+c=bcos\theta +{({r}^{2}-{b}^{2}{sin}^{2}\theta )}^{0.5},$$

where $\theta$ is the angle of incoming light. The light intensity at a distance $b$ from the reactor center is computed as follows, considering a diffuse and even illumination of the vessel from all sides:

6 $$I\left(b,X\right)=\frac{1}{\pi }\underset{0}{\overset{\pi }{\int }}I\left(b,\theta ,X\right)\mathrm{d}\theta =\frac{{I}_{0}}{\pi }\underset{0}{\overset{\pi }{\int }}{e}^{-\alpha X(b\mathit{cos}\theta +{({r}^{2}-{b}^{2}{\mathit{sin}}^{2}\theta )}^{0.5})}\mathrm{d}\theta.$$

## Computational strategies

Equations 3 and 6 were solved numerically using a Gauss–Chebyshev quadrature formula to evaluate the integrals with respect to $b$, utilizing 100 points, and with respect to $\theta$, utilizing 40 points [16]. To determine the number of points necessary to compute the integrals, the apparent growth rate $\stackrel{-}{\mu }$ has been computed considering six cases, which are summarized in Table 1. The following parameters were chosen for the calculations: $X=20$ kg m^−3^, ${\mu }_{max}=3$ day^−1^, ${K}_{M}=5$ µmol m^−2^ s^−1^, ${I}_{0}=80$ µmol m^−2^ s^−1^, and $r=0.094$ m. We selected the high value of biomass concentration $X=20$ kg m^−3^, since it leads to relatively large deviations among the results if an adequate number of points is not chosen for the integration.

**Table 1:** Numerical results of the apparent growth rate computed using an increasing number of points to compute the integrals with respect to ${\text{b}}$ and ${\uptheta }$

Case	a)	b)	c)	d)	e)	f)
Number of points:						
$b$ $\theta$	15 5	25 10	50 20	100 40	200 80	400 160
$\overline{\mu }$ in day^−1^						
	0.0458	0.0352	0.0343	0.0339	0.03382	0.03379

The values of the growth rate decrease as the number of points increases and tend to an asymptotic value, see Table 1. However, the difference between the growth rate computed between Case d) and Case e) is approximately 0.24%, while the one computed between Case e) and Case f) is approximately 0.089%. Therefore, we conclude that the number of points used in Case d) is sufficient to compute the integrals in Eqs. 3 and 6 with enough accuracy.

The growth of the culture was calculated by solving numerically Eq. 2 combined with Eq. 14 utilizing a first-order forward Euler method. To justify the choice of the time integration method, the results obtained with the classical first-order forward Euler method were compared with those obtained with the Runge–Kutta method of the subroutine ODE45 implemented in MATLAB 2017^®^. Growth data similar to those of *Arthrospira platensis* were used with ${\mu }_{max}=3$ day^−1^, ${K}_{M}=50$ µmol m^−2^ s^−1^, ${I}_{0}=80$ µmol m^−2^ s^−1^, and $r=0.0495$ m, see Table 5.

**Fig. 2:**
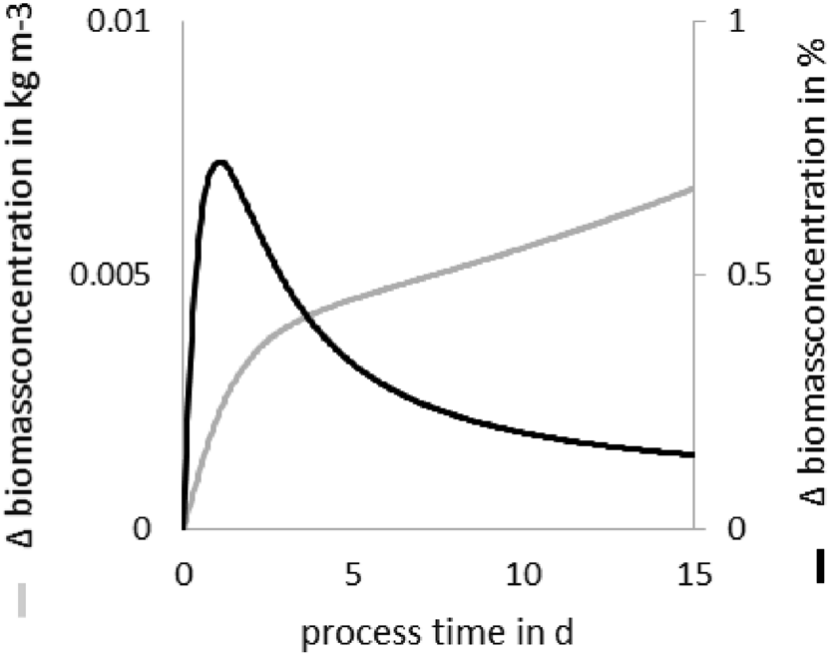
Difference between the numerical results obtained with the first-order Euler method and the Runge–Kutta method of the function ODE 45. The light gray line indicates the absolute difference and the black line the percentage differences between the results

Figure 2 displays the difference (absolute and percentage) of the outcome obtained with both methods. The cultivation period is 15 days, and the time step is fixed to 1 h. The L-infinity norm is ${L}_{\infty }=\mathrm{max}\left(\left|{X}_{iRK}-{X}_{iE}\right|\right)=3.8\cdot {10}^{-3}$ kg m^−3^, where ${X}_{iRK}$ and ${X}_{iE}$ are the biomass concentrations at a time step $\mathrm{i}$ computed with the Runge–Kutta and the Euler methods, respectively. Since the L-infinity norm is small, it can be concluded that the difference between the results of both numerical schemes is negligible. Therefore, we retain the use of the first-order Euler method to integrate Eq. 2 in all our numerical computations.

## Derivation of the growth kinetics

In the previous two sections, we outlined the theoretical and computational strategies deployed when calculating the growth rate in photobioreactors via a standard light attenuation model. We use these calculations later in this paper to calibrate the new model we are proposing. However, the derivation of growth kinetics achieved by our model is independent of those described in “Theory”.

**Scheme 1:**
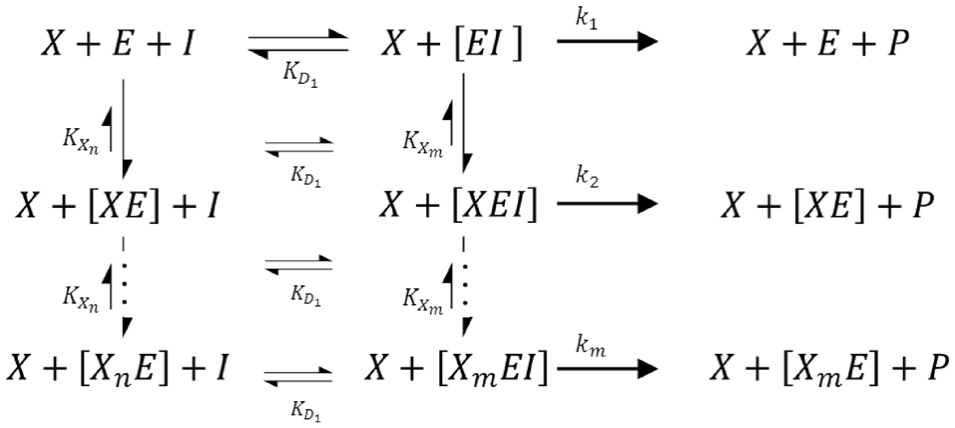
Reaction scheme for the kinetic-based model of the effect of biomass on the apparent growth of a culture

The basic principle of our approach for estimating the apparent growth inside photobioreactors consists of simple enzyme kinetic models. Even though the surrounding biomass $X$ does not directly bind to the active center of the photosynthesis $E$, we conceive of the surrounding biomass as an inhibitor for photosynthesis. The reaction equation in Scheme 1 illustrates possible influences of the surrounding biomass on the light-dependent kinetics inside the cell.

The left side of the scheme represents the self-shading effect of the biomass which directly interacts with the reaction between $E$ and the light $I$. $E$, in this case, could be understood as the photosynthetic unit (PSU), and $I$ as a substrate, which would be in that case a photon. In this sense, the traditional enzyme–substrate complex $\left[EI\right]$ could describe an activated PSU (PSU*). For correctness, it should be mentioned that if $I$ represents a photon, the reaction between $E$ or $\left[EI\right]$ would not be considered an equilibrium reaction, and the equilibrium constant would change to a reaction constant ${K}_{{D}_{1}}=\frac{1}{K}$ [10]. However, the subsequent derivations will remain the same. A further postulation is that not only one single cell is affecting an adjacent cell. Hence, the influence of multiple cells is taken into account by an interaction coefficient $n$ on the left side of the equation, and a coefficient $m$ for the interaction of the biomass with the $\left[EI\right]$ on the right side of the equation. Considering a pseudo first-order reaction, the biomass itself is not influenced by the amount of the assumed complexes with $E$ or $\left[EI\right]$. Additional assumptions state that the reaction between the inactivated enzyme and the enzyme–substrate complex is independent of the degree of influencing biomass (represented by the parameters $m$ and $n$) and that the position of the equilibrium lies on the side of the biomass inhibition. Keeping the association to the PSU, $P$ could be understood as a product of the light cycle (e.g., ATP or NADPH), and, therefore, could be related to the growth rate. The authors want to stress at this point that the comparison of Scheme 1 with the photosynthetic unit is just a thought model without laying any claim to correctness and completeness. Thus the concentrations ${C}_{i}$ of the complexes among $X$, $E$, $I$, and the total amount of enzyme ${E}_{T}$ can be calculated as follows, assuming steady-state conditions and simultaneous, instantaneous interaction of the surrounding biomass [17]:

7 $${C}_{\left[EI\right]}=\frac{{C}_{E}{C}_{I}}{{K}_{{D}_{1}}},$$

8 $${C}_{\left[{X}_{n}E\right]}=\frac{{C}_{E}{\left({C}_{X}\right)}^{n}}{{\left({K}_{{X}_{n}}\right)}^{n}},$$

9 $${C}_{\left[{X}_{m}EI\right]}=\frac{{C}_{\left[EI\right]}{\left({C}_{X}\right)}^{m}}{{\left({K}_{{X}_{m}}\right)}^{m}},$$

10 $${C}_{{E}_{T}}={C}_{E}+{C}_{\left[EI\right]}+{C}_{\left[{X}_{n}E\right]}+{C}_{\left[{X}_{m}EI\right]}.$$

The steady-state conditions at equilibrium can be inserted into the following function of the general reaction kinetics for mixed inhibitions:

11 $$\stackrel{-}{\mu }\;=\;{C}_{\left[EI\right]}{k}_{1}+{C}_{\left[{X}_{m}EI\right]}{k}_{m},$$

where $\stackrel{-}{\mu }$ is the apparent average growth rate observed in the reactor systems. Inserting Eqs. 7 and 9 into Eq. 11, and expressing ${C}_{E}$ by substituting Eqs. 7–9 into Eq. 10, it results in the following logistic function:

12 $$\stackrel{-}{\mu }=\frac{{\mu }_{max}\left(1+\frac{{k}_{m}}{{k}_{1}}{\left(\frac{{C}_{X}}{{K}_{{X}_{m}}}\right)}^{m}\right)}{\frac{{K}_{{D}_{1}}}{{C}_{I}}\left(1+{\left(\frac{{C}_{X}}{{K}_{{X}_{n}}}\right)}^{n}\right)+1+{\left(\frac{{C}_{X}}{{K}_{{X}_{m}}}\right)}^{m}},$$

where ${\mu }_{max}$ is the theoretical highest achievable growth rate using the total amount of enzymes ${C}_{{E}_{T}}$.

13 $${\mu }_{max}={k}_{1}{C}_{{E}_{T}}.$$

Equation 12 is a mixed-inhibition kinetic formula, where the inhibitor has cooperative properties, similar to the Langmuir-Hill equation. The main difference to a Langmuir-Hill equation is the use of two different “Hill-coefficients” ($m$ and $n$), which extends the sigmoidal function with asymmetric properties. The parameters required as input for predicting the growth are themselves dependent on the light supply and the reactor geometry. These parameters are calibrated using the numerical solutions of Eqs. 3 and 6 for different incident light intensities and reactor diameters. This procedure of finding suitable regression curves for every single parameter is described in the section Results and discussion.

## Materials and methods

### Organisms

Three different phototrophic organisms were used to verify the models presented in this paper. The algal strain *Chlamydomonas asymmetrica* is a self-isolate from freshwater in South Korea. The red algae *Porphyridium purpureum* (CCAP 1380/3), as well as the Cyanobacterium *Arthrospira platensis* (NIES-39), were obtained from culture collections.

### Cultivation

For cultivation, three cylindrical photobioreactors with different radii $r=0.033$ m, $r=0.0495$ m, and $r=0.094$ m were used. The cultivation took place after the sterilization of the reactor systems. Fluorescent lamps (Lumilux Cool White L 18 W/84, FA. Osram) were used as a light source, the light intensity varied with the experimental setup and the used intensities are noted within the description of the results. The light supply was continuous over the whole cultivation time. The light intensity for an experiment was adjusted using a Quantum light sensor (Li-250A, Li-Cor US), and it was measured on the surface of every reactor in different places. Afterward, it was averaged. The culture conditions and used media for the different algae are given in Table 2. The water of the media was autoclaved, and after the preparation of the media sterile filtrated through a 0.22-µm Millipore filters.

**Table 2:** Culture conditions for the different organisms

Organisms	Temperature (°C)	Media
*Chlamydomonas asymmetrica*	30	AF6 (Kato, 1982)
*Porphyridium purpureum*	20	artificial seawater (Jones et al., 1963)
*Arthrospira platensis*	30	SOT (Ogawa & Terui, 1970)

CO_2_ gas was added to the aeration (3%) whereas a total gas flow rate of 0.5 vvm was used and the gas was filtered before entering the reactor using a 0.22-µm air filter from Sartorius. For the determination of the growth, the biomass concentration was measured indirectly via extinction at 750 nm and directly via lyophilized dry weight.

## Results and discussion

### Description and final form of the new logistic formula

The derived Eq. 12, which is based on the enzymatic model illustrated in Scheme 1, shows the dependency of the apparent growth rate of culture on the biomass concentration. The calibration of the empirical constants (${K}_{{D}_{1}}$, ${K}_{{X}_{m}}$, ${K}_{{X}_{n}}$, $\frac{{k}_{m}}{{k}_{1}}$, ${\stackrel{-}{\mu }}_{max}$, $m$*, *$n$) in Eq. 12 is based on the numerical solution of Eqs. 3 and 6. A simplification can be achieved by combining the half-saturation constant ${K}_{M}$ with the incident light intensity ${I}_{0}$. The numerical solutions using the same ratio between incident light intensity and ${K}_{M}$ are identical, hereafter $L=\frac{{I}_{0}}{{K}_{M}}$ will be used. To perform the calibration, values of ${K}_{M}$ and ${\mu }_{max}$ are identical to those used for calculating the numerical data.

Before performing the calibration, we investigated how different values of incident light intensities ${I}_{0}$, half-saturation constants ${K}_{M}$, and reactor radii $r$ influence the growth rate. To this end, values of different light intensities ranging between ${I}_{0}=1$ and ${I}_{0}={10}^{4}$ µmol m^−2^ s^−1^ were used in combination with different reactor sizes, ranging from a radius of $r=0.01$ m to $r=0.2$ m, and ${\mu }_{max}=0.6$ day^−1^.

**Fig. 3:**
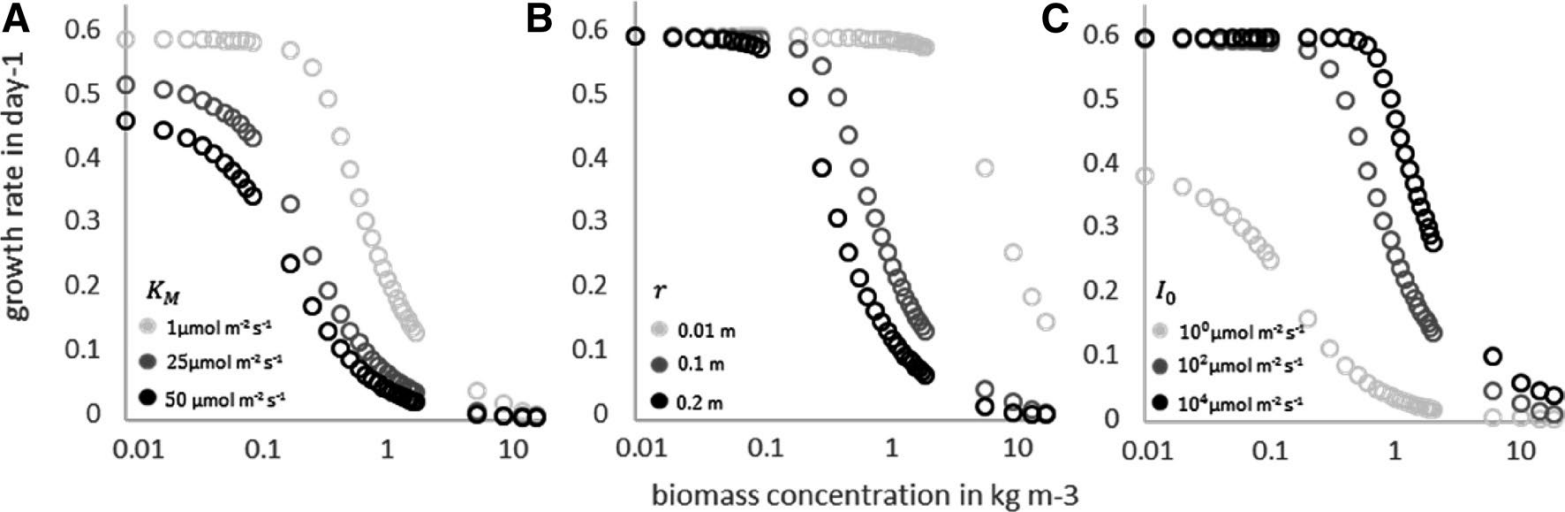
Growth rate versus biomass concentrations graphs for different settings of the parameters: **a** variation of $K_{M}$,**b** variation of reactor radius, and **c** variation of light intensity

The influence of the variation of those parameters on the growth rate is shown in Fig. 3. As mentioned before, the influence of ${K}_{M}$ and ${I}_{0}$ is similar: at low biomass concentration low values of ${K}_{M}$ and high values of ${I}_{0}$ lead to the maximum growth rate (${\mu }_{max}$), whereas high values of ${K}_{M}$ and low values of ${I}_{0}$ result in low growth rates due to light limitation, see Fig. 3a, c. The radius ($r$) does not influence the maximum achievable growth rates, but it enhances self-shading effects; a bigger radius leads to limiting self-shading effects at lower biomass concentrations, see Fig. 3b. This brief analysis concerning the effects of varying ${I}_{0}$, ${K}_{M}$, and $r$ on the growth rate suggests the use of an asymmetric sigmoid function. The fitting of Eq. 12 to the numerical data has been performed with SigmaPlot. To perform the calibration, we did not use the values of the light intensity, but we utilized the dimensionless parameter $L$. The used value of $L$ might be unusually high, but the ratio of ${I}_{0}$ and ${K}_{M}$ makes those high values necessary especially at low ${K}_{M}$ values.

**Fig. 4:**
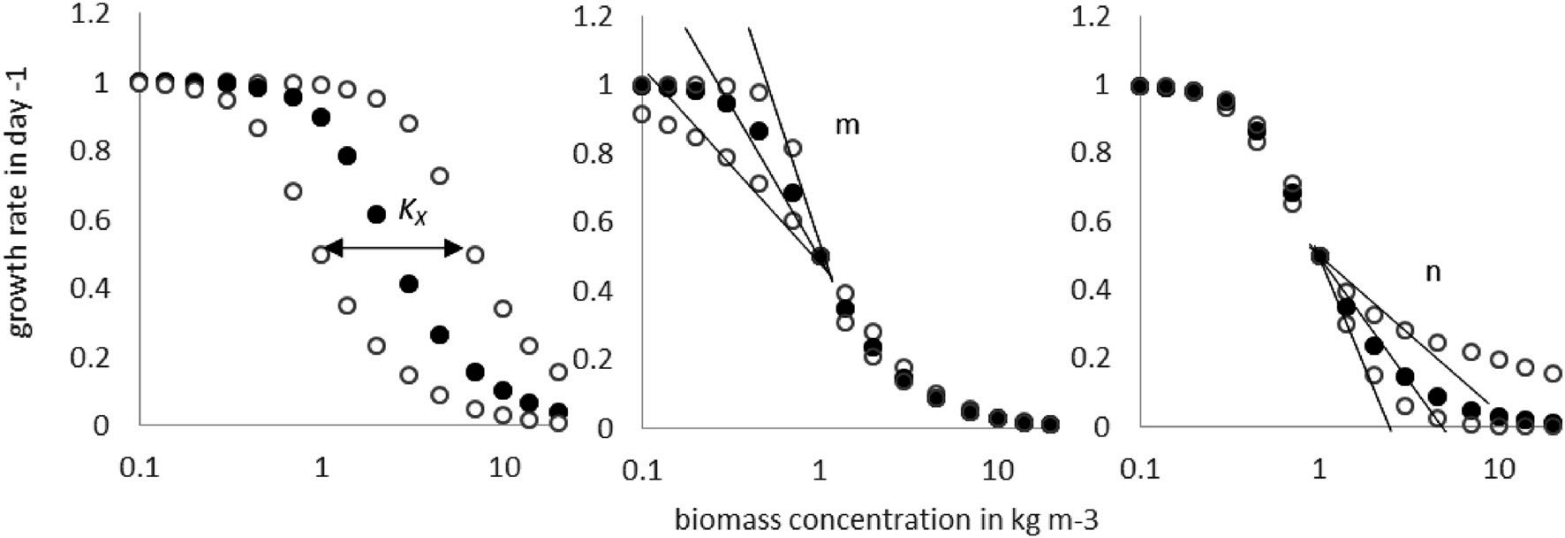
Influences of the parameters ${\text{K}}_{{\text{X}}}$*,*${\text{ m}},$ and ${\text{n}}$ of Eq. 14 on the shape of the curve

The parameters of Eq. 12 have two different properties. The values ${K}_{Xm}$ and ${K}_{Xn}$ are responsible for a shift of the curve along the axis of the biomass concentration, whereas the exponents *m* and *n* determine the steepness of the function, see Fig. 4. ${\stackrel{-}{\mu }}_{max}$ is simply a factor of the whole equation and gives the maximum growth rate of the culture for certain ${I}_{0}$ and ${K}_{M}$ constellations, and it can be read out at very low biomass concentrations, where the self-shading effects are negligible. For the simplification of the fitting, we replaced ${K}_{{X}_{m}}$ and ${K}_{{X}_{n}}$ with ${K}_{X}$, and additionally, we set $\mathrm{Formula}=\frac{{K}_{{D}_{1}}}{{C}_{I}}$. Since the first results indicated that the quota $\frac{{k}_{m}}{{k}_{1}}=1$, this ratio was fixed for all performed fittings. Equation 12 is then modified to its final version:

14 $$\stackrel{-}{\mu }=\frac{{\stackrel{-}{\mu }}_{max}\left(1+{\left(\frac{{C}_{X}}{{K}_{X}}\right)}^{m}\right)}{S\left(1+{\left(\frac{{C}_{X}}{{K}_{X}}\right)}^{n}\right)+1+{\left(\frac{{C}_{X}}{{K}_{X}}\right)}^{m}}.$$

### Calibration of the parameters of the new logistic function

Independent of the parameter settings, the correlation between the numerical data and the fitting function has at least ${R}^{2}=0.9952$ and the sum of squared residuals (RSS) is lower than 0.0334. The parameter setting of the worst fit corresponds to $L=100$ and the reactor radius is $r=0.05$ m.

The resulting values show that the exponents $m$ and $n$ are independent of the other parameters in the formula. On the contrary, the values of $S$ and $K_{X}$ affect each other. Moreover, it was found that the factor $S$ can be expressed as a function of light intensity which can be described by a hyperbolic function:

15 $$S = \frac{L + S_{1}}{S_{2}L},$$

with $R^{2} = 0.996$. The resulting values of the performed calibration of the parameters of Eqs. 15, 16, 17, 19, and 20 are listed in Table 3 in the column named “Value”.

**Table 3:** Sensitivity of Eq. 14 to OAT variation of the input parameters to achieve at least $R^{2} = 0.99$

Parameter	Light regime of the highest sensitivity (L)	Value	${\varvec{R}}^{2} > 0.99$				
			Upper limit	Lower limit	Deviation	Deviation in %	Average deviation
${\varvec{S}}$		Equation 15					
$S_{1}$	1	75.4	120.98	46.72	0.0585	58.20	28.72
$S_{2}$	independent	1.9	3.042	1.04	0.0585	58.20	28.72
${\varvec{K}}_{{\varvec{X}}}$		Equations 16 & 17					
$K_{{X_{1} }}$	1	0.2	0.304	0.133	0.0571	46.571	23.85
$K_{{X_{2} }}$	1	0.443	0.762	0.271	0.0571	46.572	23.85
$K_{{X_{3} }}$	1000	0.5	0.740	0.355	0.126	28	7.55
$K_{{X_{4} }}$	1000	0.0005	0.00125	0	0.135	40.373	8.189
${\varvec{n}}$		Equation 19					
$n_{1}$	1000	1.27	3.50	0.495	0.219	181.91	20.34
$n_{2}$	1	2.68	214.1	0	0.057	24.35	12.82
${\varvec{m}}$		Equation 20					
$m_{1}$	1000	2.4	6.97	0.646	0.095	11.34	3.85
$m_{2}$	1	56.6	403	10	0.0590	27.95	12.32

By substituting Eq. 15 and the quota $\frac{{k_{m} }}{{k_{1} }} = 1$ into Eq. 12, a better overall fit to the numerical data is obtained, where the lowest $R^{2} = 0.9984$ at $L = 5 \times 10^{5}$ and $r = 0.01$ m.

The values of $K_{X}$ show a very distinct dependency on the radius of the reactor and they can be expressed as:

16 $$K_{X} = \frac{A}{r},$$

with $R^{2} = 0.9962$ and a standard error of estimate $SE = 0.3384$. The parameter $A$ depends on the light intensity. The value of $A$ first decreases until $L \approx 100$ and then increases at higher light intensities. The reciprocal values of $A$ follow a trend similar to light inhibition and can be fitted with a standard formula for substrate inhibitions:

17 $${{A}} = { }\frac{{{{K}}_{{{{X}}_{1} }} }}{{\frac{{{{K}}_{{{{X}}_{2} }} }}{{{{K}}_{{{{X}}_{2} }} + \frac{2}{{{L}}}}} + \frac{1}{{{{K}}_{{{{X}}_{3} }} + {{K}}_{{{{X}}_{4} }} {{L}} + \frac{1}{{{{K}}_{{{{X}}_{2} }} {{ L}}}}}}}},$$

which gives $R^{2} = 0.9023$ and $SE = 0.197$. The combined influence of $r$ and $L$ on $K_{X}$, by substituting Eq. 17 into Eq. 16 leads to $R^{2} = 0.9705$. For $L \ge 100$ and $L < 100$ a distinct correlation can be found between $K_{X}$ and biomass concentration at which the culture growth is $\overline{\mu }_{max} /2$. This value could be used for the comparison of different reactor types, as it provides information about how the reactor geometry inhibits the growth of algae.

The parameters $m$, $n$, and $\overline{\mu }_{max}$ can be regarded to be independent of the reactor size and they achieve a constant value at high light intensities. Therefore, they can be fitted using hyperbolic formulas in the form proposed by Michaelis and Menten:

18 $${\overline{\mu }}_{{{\text{max}}}} = \frac{{{\upmu }_{{{\text{max}}}} {{ L}}}}{{1 + {{L}}}},$$

19 $$n = \frac{{{{n}}_{1} {{ L}}}}{{{{n}}_{2} + {{L}}}},$$

20 $$m = - \left( {1 + \frac{{{{m}}_{1} {{ L}}}}{{{{m}}_{2} + {{L}}}}} \right).$$

The fit of Eq. 18 to the values of $\overline{\mu }_{max}$ depicts a correlation of $R^{2} = 0.9999$ independent of the radius of the reactor. For $m$ and $n$, the quality of the correlation between values of Eq. 19 and Eq. 20 as well as the results of the regression with SigmaPlot varies depending on the radius. Generally, reactors with bigger diameters exhibit $R^{2}$ closer to 1 compared to reactors with smaller diameters. The lowest $R^{2}$ for $n$ is $R^{2} = 0.15$ for a small reactor with $r = 0.01$ m. This is due to an insufficient determination of $n$ at $L$ above 10,000. Only considering lower light intensities shows a correlation with $R^{2} = 0.87$.

The calibration reduces the number of required inputs for the model equation to four. Two are process-related ($I_{0}$, $r$) and two are empirical ($\mu_{max}$, $K_{M}$). Although this might appear comparable to a Type I model according to the categorization of Béchet at first sight, it is not [12]. The main difference is its inherent adaptiveness to the change of process parameters ($I_{0}$, $r$). This universality is only reached in standard models by averaging the local growth behavior to obtain the global growth rate in the reactor. The introduced approach obtains its general validity by a derivation of a local independent growth kinetic. Instead of correlating the influence of the local light intensity to the cell growth (inhomogeneous), the new method aims at describing this effect by inhibiting effect caused by the surrounding biomass (homogenous and, therefore, location independent). The characteristic of the inhibition was found to be predictable by the reactor radius and the incident light intensity. All the parameters used to calculate Eq. 14, except for the biomass concentration, are static values with respect to time. The parameters $S$, $A$, $\overline{\mu }_{max}$, $m$, and $n$ in Eqs. 15, 17, 18, 19, and 20 are not only independent of time but also the radius of the cylindrical reactors and biomass concentration. Besides, the parameters $S_{1}$, $S_{2}$ $K_{{X_{1} }}$, $K_{{X_{2} }}$, $K_{{X_{3} }}$, $K_{{X_{4} }}$, $n_{1}$, $n_{2}$, $m_{1} ,$ and $m_{2}$ listed in Table 3 are constant values obtained from the calibration and never change. Consequently, the new logistic formula introduces a stable, universal way to calculate growth rates, only depending on the process parameters $I_{0}$, $r,X,$ and the empirical parameters $\mu_{max}$ and $K_{M}$.

### Sensitivity analysis

For estimating the sensitivity of Eq. 14, the input parameters of Eqs. 15, 17, 19, and 20 are varied one-at-a-time (OAT). The goal of this procedure is to find the lower and upper bounds of each parameter to achieve $R^{2} = 0.99$. As the influence of most of the parameters on the model sensitivity depends on the incident intensity of light, the upper and lower limits were determined for different values of $L$. Table 3 shows the intervals for every single parameter, the resulting maximum deviation, the percentage deviation, and the average deviation calculated for biomass concentrations between 0.01 and 20 kg m^−3^ for the light regime with the highest sensitivity.

Variations of the parameter $K_{{X_{4} }}$ have the lowest influence on the outcome of Eq. 14. In the first approach of the formula, this parameter was implemented to enable the fitting to a very high value of $L$, that is, $L \approx 10^{4}$. Since, in experiments, those scenarios are very rare—only achievable for $K_{M} < 1$ — the term $K_{{X_{4} }} /L$ could be excluded from the equation if used for $L < 100$. The other parameters of Eq. 17 can be altered by about ± 50%, leading to maximum deviations from the base case of 46%. The greatest deviations result from a shift of the function along the biomass concentration-axis, which leads to high variations in the steepest part of the function (see Fig. 4a). The influences of the parameters on $S$ (Eq. 15) are similar since changes also result in a shift of the function along the biomass concentration-axis.

The parameters of Eqs. 19 and 20 influence the outcome in a different way as no shift of the function occurs. The parameters change the steepness on either the left or the right side of the function. Therefore, $R^{2} = 0.99$ can be reached even with a high variation of the input parameters. Influences of Eq. 20 on the course of the whole function is confined on the right side by the value of $K_{X}$. Variations of Eq. 19 result in a widening of the function at high biomass concentrations.

Randomizing the input parameters in the intervals reported in Table 4 leads to an average $R^{2} = 0.964$, a maximal deviation of 84.5%, and an average deviation of 13.29%. If the input parameters are varied only by ± 10% much smaller deviations are found with an average deviation of 12.08%. Moreover, the highest deviations (above 30%) occur at low growth rates and high biomass concentrations. The mean percentage deviation for process-relevant biomass concentrations ($X = 0.1 - 10$ kg m^−3^) is 5.4%.

**Table 4:** Parameter range used for the comparison between numerical data obtained using Eqs. 3 and 6 and the results of the logistic function

Parameter	Range
${\varvec{X}}$(kg m^−3^)	0.01–20
${\varvec{I}}_{0}$(µmol m^−2^ s^−1^)	1–10,000
${\varvec{r}}$ *(m)*	0.01–0.2
${\varvec{K}}_{{\varvec{M}}}$(µmol m^−2^ s^−1^)	1–10

### Comparison of the new model with numerical data

**Fig. 5:**
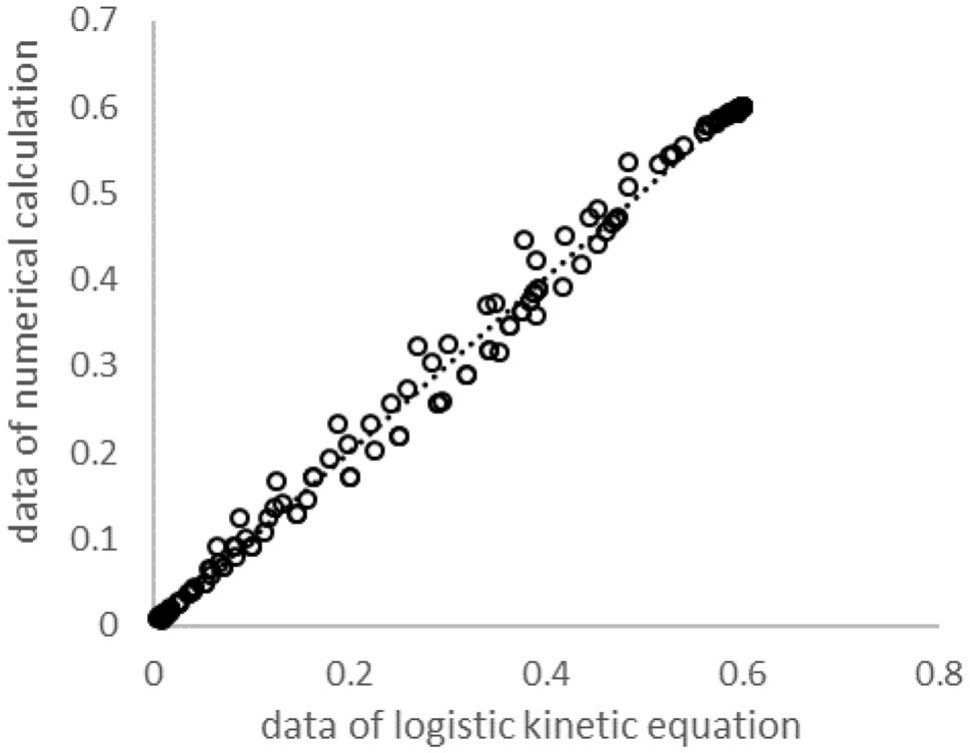
Comparison between numerical data (Eqs. 3 and 6) and the results of the logistic equation (Eq. 14) of ${\overline{\mu }}$ for more than 150 different parameter settings

Figure 5 shows the comparison of the results of the calibrated Eq. 14 to numerical data calculated using Eqs. 3 and 6. The comparison considers 150 variations of $$X$$, $$I_{0}$$, $$r$$$$,$$ and $$K_{M}$$ values. The $$SE$$ between the logistic equation and the numerical data is 0.0146 and has an adjusted $$R^{2} = 0.9948$$. Therefore, the new formula can be regarded as a very good approximation to the numerical calculation of Eqs. 3 and 6, for different incident light intensities and reactor diameters. Models based on the average light intensity, which are not calculating the average local growth rate in the reactor, cannot be used to fit numerical solutions — as described in the introduction.

## Experimental verification

The final step in the verification of the new approach is a direct comparison of the model with experimental data using three representatives of photosynthetic organisms from different taxonomic groups. In batch cultivation systems, both biomass and biomass-dependent inhibiting effects are continuously increasing with culture duration. According to our model, this increase in biomass concentration is the only reason for the decrease of culture growth, if other culture parameters are kept constant and no limitation of nutrients occurs. The cultivation took place in three cylindrical reactors with different diameters. The light came from four directions, and both the incident light intensity and the temperature were kept constant during the cultivation. Each batch cultivation was carried out in triplicates and is represented as an average value. A continuous light has been applied during the cultivation time to test the model on the proposed influence of the inhibiting effect of the biomass.

The simulations of the growth rate are always performed with the constant parameters listed in Table 3 and with the parameters $S$, $A$, $\overline{\mu }_{max}$, $m$, and $n$, which only depends on $I_{0}$, $r,$ and the used microalgae ($K_{M}$). They are listed in Table 5 for different microalgae and process conditions. The only parameters that must be adjusted for different algae are the empirical parameters $\mu_{max}$, $K_{M} ,$ and $\dot{D}$. Those empirical parameters themselves are independent of the process parameters $r$ and $I_{0}$, as light inhibition effects are not considered at this stage of the model. Equation 2 was first computed numerically without including the respiration decay rate $\dot{D}$. This allowed identifying the values of $\mu_{max}$ and $K_{M}$ focusing on a good fit at the beginning of the culturing process, i.e., in the light saturation regime, and the transition to the light-limited regime.

**Table 5:** Process and empirical parameters used for the simulation of the growth behavior of *C. asymmetrica*, *P. purpureum*, and *A. platensis* in different cylindrical reactors

Algae	${\varvec{\mu}}_{{{\varvec{max}}}}$	${\varvec{K}}_{{\varvec{M}}}$	$\dot{\user2{D}}$	${\varvec{I}}_{0}$	${\varvec{r}}$	${\varvec{S}}$	${\varvec{K}}_{{\varvec{X}}}$	$A$	${\stackrel{-}{\mu }}_{max}$	$n$	${\varvec{m}}$	R^2^	*SE*	R^2^	*SE*
Origin of values	Empirical			Process defined		Calculated using the equation below and constants in Table 3						Figure 6		Figure 7	
						Equation 15	Equation 16	Equation 17	Equation 18	Equation 19	Equation 20				
*C. asymmetrica*	7	100	0.01	360	0.033	11.55	4.56	0.15	5.48	0.73	− 1.14	0.966	0.24	0.966	0.407
				360	0.0495	11.55	3.07	0.15	5.48	0.73	− 1.14	0.985	0.081	0.928	0.197
				290	0.094	14.21	1.81	0.17	5.20	0.66	− 1.12	0.937	0.085	0.964	0.068
*A. platensis*	1.5	46.8	0.02	250	0.033	25.33	7.72	0.25	1.85	0.47	− 1.07	0.980	0.382	0.916	0.082
				250	0.0495	25.33	5.20	0.25	1.85	0.47	− 1.07	0.943	0.280	0.997	0.014
				250	0.094	25.33	2.71	0.25	1.85	0.47	− 1.07	0.996	0.010	0.992	0.037
*P. purpureum*	1	10	0.01	250	0.033	1.15	2.25	0.074	0.79	1.22	− 2.27	0.989	0.497	0.972	0.038
				250	0.0495	1.15	1.51	0.074	0.79	1.22	− 2.27	0.998	0.123	0.979	0.065
				250	0.094	1.15	0.79	0.074	0.79	1.22	− 2.27	0.997	0.102	0.982	0.015

**Fig. 6:**
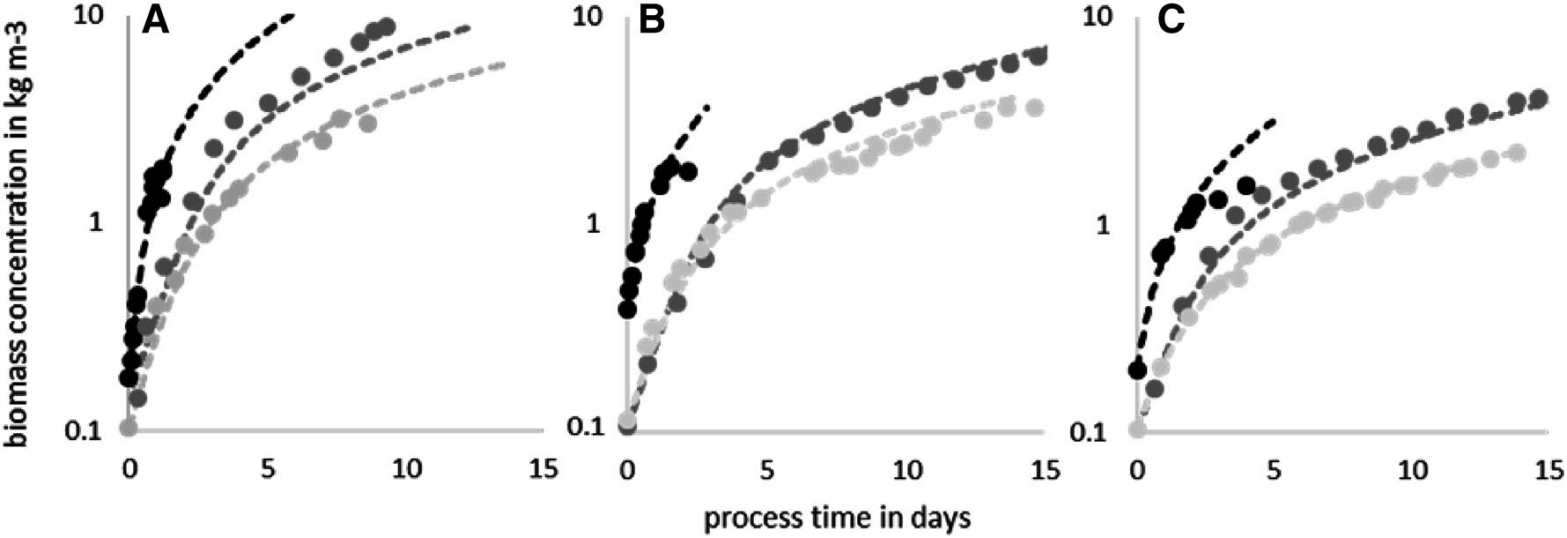
Growth behavior of three different organisms (black C. asymmetrica; dark gray P. purpureum; light gray A. platensis) in three reactors of different sizes (**A**: ${\text{r}} = 0.033{ }$ m, **B**: ${\text{r}} = 0.0495{ }$ m, **C**: ${\text{r}} = 0.094{ }$ m). The symbols represent experimental data, and the dashed lines are the numerical results obtained using Eq. 2 in combination with Eq. 14

However, most of the time this resulted in an over-prediction of the biomass concentration in the advanced stage of the cultivation (light limitation due to high biomass concentrations). Hence, after obtaining suitable values of $\mu_{max}$ and $K_{M}$, a constant respiration decay rate $\dot{D}$ was subsequently included in the numerical solution of Eq. 2, to further improve the agreement of the model to the experimental data, see Table 5. Figure 6 shows a comparison between the experimental data and the results of the simulation. All the experiments show the effect of a decreasing growth rate with an increase in biomass concentration. Increasing the reactor size leads to an earlier decrease in the growth rates and peak cell densities, as expected.

As far as the apparent growth rate concerns, the green algae *C. asymmetrica* is the fastest of the tested algae, with an apparent growth rate of 2.4 per day (in the smallest reactor at low cell densities). The best match between simulation and experiments was achieved with $\mu_{max} = 7$ day^−1^ and $K_{M} = 100$ µmol m^−2^ s^−1^. At the beginning of the cultivation, the simulation and the experimental outcome were in good accordance. In the small ($r = 0.033$ m) and the middle-sized ($r = 0.0495$ m) reactor, the culture died right after the growth phase before reaching a stationary, light-limiting phase, see Fig. 6a, b. The culture break-in could be caused by a sudden depletion of nutrients in the media. In the biggest reactor ($r = 0.094$ m), the culture reached slightly lower biomass concentrations compared to those predicted in the light-limiting phase of the cultivation, see Fig. 6c. In this case, it is more likely that the mass transport of CO_2_ is not sufficient to supply higher biomass concentrations, which could lead to smaller growth rates or even result in cell death, other than the uniform death rate.

The culture growth of the other two algae does not show any limitations coming from dissolved nutrients or mass transport phenomena. The reason could lie within the different media compositions and the slower metabolism, according to the lower growth rates.

The growth of the *A. platensis* cultures can be best simulated using a $\mu_{max} = 1.5$ day^−1^ and $K_{M}$ = 46.8 µmol m^−2^ s^−1^. Considering the difference in cultivation temperature, the empirical growth values are similar to those used by Cornet et al*.* or Levert et al. for their model [18, 19]. The worst fit is achieved for *A. platensis* in the reactor with $r = 0.0495$ m ($R^{2} = 0.943$ and $SE = 0.280$). A direct comparison of the growth behavior between *P. purpureum* and *A. platensis* shows that *P. purpureum* has a smaller growth rate than *A. platensis.* However, the latter has a higher value of $K_{M}$, and, therefore, a lower affinity to light. This indicates that *A. Platensis* needs more light to reach half of the maximum growth. Therefore, *A. platensis* grows faster than *P. purpureum* at the beginning of the cultivation process. However, the growth of *A. platensis* decreases faster than the one of *P. purpureum* in a later stage of the cultivation, due to the higher self-shading effects that occur because of a higher necessity of light (higher $K_{M}$ values). This effect is visible in the reactors with radii $r = 0.033{ }$ m and $r = 0.0495{ }$ m. In the biggest reactor ($r = 0.094$ m), *A. platensis* is already light-limited at the beginning of the cultivation process and the growth of both species is approximately equal. However, the growth of *A. platensis* is smaller than the one of *P. purpureum* at a later stage of cultivation due to its higher necessity of light. The growth of *P. purpureum* can be best predicted using $K_{M} = 10$ µmol m^−2^ s^−1^ and $\mu_{max} = 1$ day^−1^ with the highest $SE = 0.497$ for the reactor with size $r = 0.033$ m.

**Fig. 7:**
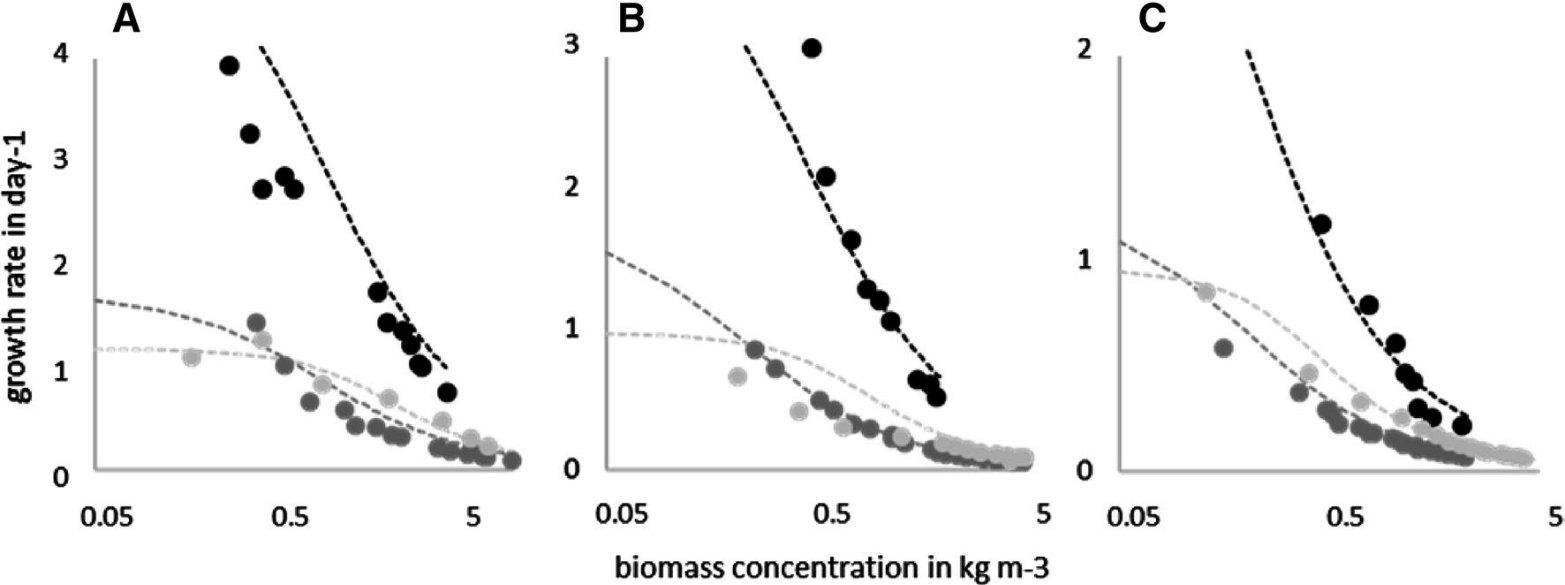
Growth rate as a function of biomass concentration for different organisms (black C. asymmetrica; dark gray P. purpureum; light gray A. platensis) in three reactors of different sizes (**A**: ${\text{r}} = 0.033{ }$ m, **B**: ${\text{r}} = 0.0495{ }$ m, **C**: ${\text{r}} = 0.094{ }$ m). Symbols represent experimental data, and the dashed lines represent results obtained using Eq. 14 with the constant parameters listed in Table 3

To elucidate the need for an asymmetric logistic function, the results of the culture development displayed in Fig. 7 are illustrated considering the growth rate as a function of the biomass concentration.

This way of representing the data shall accentuate that the decrease of culture growth with increasing cultivation time is due to the self-shading effect, which in the new model is taken into account by the inhibiting effect of the surrounding biomass. The R^2^ between the model and the experimental data are above 0.93, and, therefore, of similar goodness as those described before for the numerical simulation of the culture growth over time (Fig. 6).

The new model shows an alternative way of approaching the calculation of biomass growth inside photobioreactors, and it can be improved similarly as light attenuation models evolved from Type I to Type III. The parameters of Eq. 14 were calibrated to a growth model, which only considers the adsorption of light (Eq. 4), and the correlation between light intensity and growth rate, according to a simple Monod kinetic (Eq. 3). However, the asymmetrical, highly adjustable nature of Eq. 14 allows modifications to include other light attenuation models (see Fig. 3). For example, light inhibition effects would cause a change in Eq. 18 and the implementation of light scattering could lead to a change of Eq. 19, as scattering effects are more dominant at high biomass concentrations ($X > 3$ kg m^−3^) [20]. For biological effects, like light-acclimation, where the amount of pigments changes over the process as a function of the available light and the light history of the cell, the parameters of the new logistic function need a new calibration concerning all new influencing parameters. With the change of the content of the pigments, besides the adsorption, the affinity of the algae towards lights of different wavelengths changes. A possible extension of the new model would incorporate those effects, describing the inhibiting effects of the biomass for each wavelength and pigment composition.

As far as other limiting factors concern, the new model can also be extended and modified, as it has been done with other models. In so-called uncoupled models, the effect of temperature is seen as independent of light [12]. The influence of temperature could be accommodated by multiplying the resulted growth rate with the Arrhenius equation or more advanced models, which take into account the negative effect of high temperatures on enzymatic reactions and cell growth [21, 22]. The effects of depletion of nutrients and limitations of mass transfer could be considered similarly as a Monod equation with multiple substrates does, by multiplying a logistic term for each considered substrate.

Although most culture processes and reactor systems do not operate in photo-inhibited conditions, the effect of photoinhibition can also be included in the new model to widen its applicability to situations where due to high light intensities photoinhibition cannot be avoided. An example is represented by outdoor cultivation systems. However, the present model is based on a kinetic function that already considers the inhibition of the surrounding biomass. Therefore, the presence of an extra term that considers photoinhibition would have added inhibition effect, which would have made this new approach much harder to understand.

Moreover, we would like to stress again the difference between the reality and the proposed model. In a biological sense, the biomass growth of phototrophic algae depends on the local light intensity inside the reactor, which is dependent on the surrounding biomass due to light absorption and scattering processes. However, the resulting inhibition kinetic formula, that is, Eq. 14 permits the computation of the growth rate of the culture without the need of calculating the local light intensity. The new model necessitates only the incident light intensity $I_{0}$ and the reactor radius $r$ as input parameters. In a common type II model, one needs to compute the local light intensity inside the reactor and the growth rate by integrating over either surfaces or volumes, while in the new logistic model no local information is required. Consequently, the end-user requires much less knowledge about a specific system, since it is already incorporated in the parameters of the new logistic function which have been calibrated over a wide range of reactor radii ${\text{ r}}$, incident light intensities $I_{0}$, and $K_{M}$ values.

## Conclusion

We proposed a new logistic equation as an alternative model for the calculation of the apparent growth rate in cylindrical photobioreactors. Different from other models, the new approach uses a biomass inhibition concept (mixed inhibition) instead of calculating the light attenuation inside the reactor. Solving for the inhibition kinetics results in an asymmetric sigmoid relation between biomass concentration and apparent growth rate. We were able to express the parameters of the new equation as a function of reactor radius and incident light intensity. The resulting formula fits the numerical data with $R^{2} > 0.8$ for reactor radii in the range of $r = 0.01\; \text{to}\;r = 0.2$ m and light intensities up to $I_{0} = 10^{4}$ µmol m^−2^ s^−1^. The final verification of the new approach comprises the growth of three different organisms in three reactors of different diameters, resulting in $SE < 0.0478$. The authors recommend using the calibrated parameters in a range of ± 10% of the given values. The new method facilitates and simplifies the calculation of the growth of phototrophic organisms in cylindrical reactors without the need for any special numerical software. The values of the parameters of the new model, except for the biomass concentration, are static values regarding time, biomass concentration, incident light intensities, and radius if cylindrical reactors are used.

## Data Availability

All codes used during this study are available from the corresponding author upon a reasonable request.

## Abbreviations

$A$: Parameter to calculate ${K}_{X}$ (kg m^−2^)
$$a,\mathrm{c}$$: Geometric length (m)
$$b$$: Radial coordinate (m)
${C}_{E}$: Concentration of enzyme (kg m^−3^)
${C}_{\left[EI\right]}$: Concentration of Enzyme–Substrate-Complexes (kg m^−3^)
${C}_{{E}_{T}}$: Total concentration of enzyme (kg m^−3^)
${C}_{\left[{X}_{n}E\right]}$: Concentration of Enzyme- and Inhibitor- (biomass) Complexes (kg m^−3^)
${C}_{\left[{X}_{m}EI\right]}$: Concentration of Enzyme–Substrate-Inhibitor-(biomass) Complexes (kg m^−3^)
${C}_{X}$: Biomass concentration (kg m^−3^)
${C}_{I}$: Photon concentration stationary (μmol l^−1^)
$\dot{D}$: Death rate (day^−1^)
$E$: Concentration of enzyme (PSU) (kg m^−3^)
$[EI]$: Enzyme–Substrate-Complex (PSU*) (kg m^−3^)
${K}_{{D}_{1}}$: Equilibrium constant enzyme and Enzyme–Substrate-Complex (μmol l^−1^)
${K}_{M}$: Half velocity constant (μmol m^−2^ s^−1^)
${K}_{{X}_{n}}$: Equilibrium constant enzyme and inhibiting biomass (kg m^−3^)
${K}_{{X}_{m}}$: Equilibrium constant Enzyme–Substrate-Complex and inhibiting biomass (kg m^−3^)
${K}_{X}$: Replacement of ${K}_{{X}_{n}}$ and ${K}_{{X}_{m}}$ (kg m^−3^)
${K}_{{X}_{1}}$: Parameter to calculate ${K}_{X}$ (kg m^−2^)
${K}_{{X}_{2}}; {K}_{{X}_{3}};{K}_{{X}_{4}}$: Parameters to calculate ${K}_{X}$
${k}_{1}\dots {k}_{m}$: Reaction constant Enzyme–Substrate-Complexes to product (m^3^ kg^−1^)
*m*: Exponent responsible for the steepness of the function (left side)
${m}_{1}; {m}_{2}$: Parameters to calculate m
*n*: Exponent responsible for the steepness of the function (right side)
${n}_{1}; {n}_{2}$: Parameters to calculate n
$L$: Dimensionless light intensity; ratio between ${I}_{0}$ and ${K}_{M}$
$${L}_{\infty }$$: L-infinity norm
${I}_{0}$: Incident light intensity (μmol m^−2^ s^−1^)
$$p$$: Light pathway (m)
PSU/PSU*: Photosynthetic unit/ activated photosynthetic unit
$$r$$: Reactor radius (m)
RSS: Residual sum of squares
R^2^: Coefficient of determination
$s$: Distance from the reactor wall (m)
$S$: Ratio between ${C}_{I}$ and ${K}_{{D}_{1}}$
${S}_{1}; {S}_{2}$: Parameters to calculate Formula
SE: Standard error of estimate
$$V$$: Reactor volume (m^3^)
Vvm: Gas volume per Vessel volume per minute (l/min^−1^)
$\dot{V}$: Volume flow rate (m^3^ min^−1^)
${\dot{V}}_{in}$: Volume flow rate entering the reactor (m^3^ min^−1^)
${\dot{V}}_{out}$: Volume flow rate leaving the reactor (m^3^ min^−1^)
$X$: Biomass concentration (kg m^−3^)
${X}_{in}$: Biomass concentration entering the reactor (kg m^−3^)
${X}_{out}$: Biomass concentration leaving the reactor (kg m^−3^)

## Greek

$\alpha$: Absorption cross section (m^2^ kg^−1^)
${\mu }_{max}$: Maximum achievable growth rate of a single cell (day^−1^)
${\stackrel{-}{\mu }}_{max}$: Maximum achievable apparent growth rate of a culture (day^−1^)
$\stackrel{-}{\mu }$: Average apparent growth rate (day^−1^)
$\theta$: Angle of the incoming light
$$\varphi$$: Polar angle

## Declarations

### Declaration of authors’ agreement

The corresponding author Christoph Lindenberger agrees on behalf of all listed authors to the authorships and submission of the manuscript for peer review.
